# Therapeutic Potential of Natural Compounds Acting through Epigenetic Mechanisms in Cardiovascular Diseases: Current Findings and Future Directions

**DOI:** 10.3390/nu16152399

**Published:** 2024-07-24

**Authors:** Paola Bontempo, Lucia Capasso, Luigi De Masi, Angela Nebbioso, Daniela Rigano

**Affiliations:** 1 Department of Precision Medicine, University of Campania Luigi Vanvitelli, Via L. De Crecchio 7, 80138 Naples, Italy; lucia.capasso2@unicampania.it (L.C.); angela.nebbioso@unicampania.it (A.N.); 2 National Research Council (CNR), Institute of Biosciences and BioResources (IBBR), Via Università 133, 80055 Portici, Italy; 3 Department of Pharmacy, University of Naples Federico II, Via Montesano 49, 80131 Naples, Italy; daniela.rigano@unina.it

**Keywords:** cardiovascular diseases, coronary artery disease, heart failure, hypertension, myocardial infarction, vascular calcification, epigenetic changes, dietary components, plant-derived bioactive compounds

## Abstract

Cardiovascular diseases (CVDs) remain a leading global cause of morbidity and mortality. These diseases have a multifaceted nature being influenced by a multitude of biochemical, genetic, environmental, and behavioral factors. Epigenetic modifications have a crucial role in the onset and progression of CVD. Epigenetics, which regulates gene activity without altering the DNA’s primary structure, can modulate cardiovascular homeostasis through DNA methylation, histone modification, and non-coding RNA regulation. The effects of environmental stimuli on CVD are mediated by epigenetic changes, which can be reversible and, hence, are susceptible to pharmacological interventions. This represents an opportunity to prevent diseases by targeting harmful epigenetic modifications. Factors such as high-fat diets or nutrient deficiencies can influence epigenetic enzymes, affecting fetal growth, metabolism, oxidative stress, inflammation, and atherosclerosis. Recent studies have shown that plant-derived bioactive compounds can modulate epigenetic regulators and inflammatory responses, contributing to the cardioprotective effects of diets. Understanding these nutriepigenetic effects and their reversibility is crucial for developing effective interventions to combat CVD. This review delves into the general mechanisms of epigenetics, its regulatory roles in CVD, and the potential of epigenetics as a CVD therapeutic strategy. It also examines the role of epigenetic natural compounds (ENCs) in CVD and their potential as intervention tools for prevention and therapy.

## 1. Introduction

**Figure 1:**
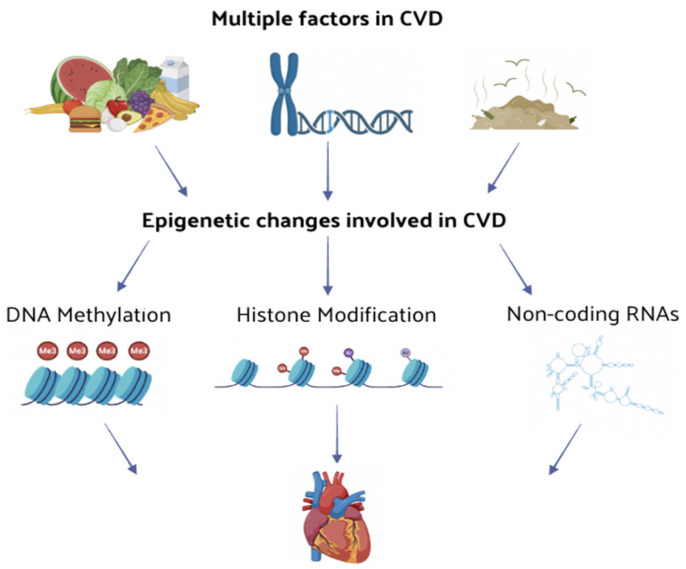
The effect of multiple factors leading to CVDs are mediated by several epigenetic changes. The function and expression level of CVD-related genes are regulated through epigenetic changes, such as DNA methylation or histone modification and non-coding RNA regulation, thus influencing the onset and progression of CVD.

Cardiovascular diseases (CVDs) are a leading cause of morbidity and mortality globally with incidence rates rising and the age of onset decreasing over the years [1]. These diseases include coronary artery disease (CAD), hypertension, heart failure (HF), myocardial infarction, and vascular calcification. Multiple biochemical, genetic, environmental, and behavioral factors can contribute to the development of CVD [2]. Recent studies highlight the significant role of epigenetic modifications in the onset and progression of CVD. Epigenetics, the primary mechanism regulating cellular responses to environmental changes, modulates genetic function, expression, and activity without altering the DNA sequence [3]. Compelling evidence suggests that epigenetic signals can induce phenotypic changes by regulating the expression of genes critical for cardiovascular homeostasis. Environmental factors influencing CVD often exert their effects through various epigenetic changes [4] (Figure 1).

Epigenetic modifications, such as DNA methylation, histone modification, and non-coding RNA regulation, significantly impact on the function and expression levels of genes related to CVD, thereby influencing its progression [5]. These changes can be relatively stable and persist over time or be reversible upon the cessation of stimuli, and crucially they are susceptible to pharmacological intervention. This opens the exciting possibility of preventing diseases by pharmacologically eliminating harmful epigenetic changes, which is a field gaining considerable attention [6]. In vitro and in vivo studies have delineated mechanisms through which various plant-derived compounds, nutrients, food components, and dietary patterns influence the epigenome [7]. Risk factors for CVD, such as obesity, inflammation, and oxidative stress, were linked to epigenetic modifications, including histone acetylation and DNA methylation. Macronutrient excesses, such as high-fat diets, or nutrient deficiencies, such as folate and other vitamins B, can influence DNA methyltransferases (DNMTs) and histone-modifying enzymes, impacting fetal growth, glucose and lipid metabolism, oxidative stress, inflammation, and atherosclerosis [8]. Bioactive compounds like polyphenols (e.g., resveratrol, curcumin, epigallocatechin) can activate sirtuins (SIRT), histone deacetylases (HDAC), or acetyltransferases, affecting inflammatory mediator responses. Many benefits of cardioprotective dietary patterns, such as the Mediterranean diet, are associated with epigenetic changes, including an altered methylation and expression of genes involved in inflammation and immune response. The development of innovative therapeutic approaches based on epigenetic changes has attracted significant attention due to the urgent clinical need for effective CVD treatments. While the impact of diet on CVD outcomes was studied in depth [8,9], the specific mechanisms through which the diet and naturally derived compounds can alter the cardiovascular epigenome still remain unknown and difficult to determine. A key research challenge is to identify which nutriepigenetic effects are reversible, enabling the translation of these findings into effective interventions for preventing or slowing the progression of CVD. In this review, we explore general epigenetic mechanisms, the role of epigenetic regulation in CVD, potential epigenetic strategies for CVD treatment, and the impact of epigenetic natural compounds (ENCs) in CVD prevention and therapy.

## 2. Epigenetic Regulatory Mechanisms

Epigenetic mechanisms modulate global gene expression without altering the underlying DNA primary structure by making covalent bonds to histone proteins and DNA double strands, which together control structure and accessibility of chromatin. These highly dynamic processes reversibly coordinate gene expression [10,11]. This reversibility highlights the critical roles that epigenetic mechanisms play in biology and suggests the potential for developing new epigenetic drugs (epi-drugs) to treat various diseases. The primary epigenetic regulatory mechanisms are described in more detail below.

### 2.1. DNA Methylation Regulation

DNA methylation is the primary form of epigenetic regulation of gene expression in mammals. This process predominantly occurs on cytosine–phosphate–guanine (CpG) sites in the promoters of genes. Cytosine methylation at these CpG islands acts as a repressive mechanism, affecting the chromatin structure and gene transcription [12]. The regulation of DNA methylation through DNMTs is essential for transferring epigenetic information to offspring. These key enzymes in DNA methylation are categorized based on their different roles as writers, erasers, and readers. Writers, such as DNMT1, DNMT3a, and DNMT3b, catalyze the addition of methyl groups to cytosine residues. These enzymes can interact with components of transcription factor complexes or repressors to target specific DNA regions for methylation [13]. Erasers modify the DNA methylation status by removing methyl groups, thereby reversing the methylation marks. Readers recognize and bind to methylated DNA, influencing gene expression. These reader enzymes play a crucial role in recruiting factors involved in DNA metabolism (replication, transcription, recombination, and repair). DNA methylation is recognized by three main protein families: the Methyl-CpG Binding Domain (MBD) family, which has a conserved MBD with high binding affinity for methylated CpG sites, recognizing methylated CpGs and repressing transcription [14]; the Ubiquitin-like with Plant Homeodomain (PHD) and Interesting New Gene (RING) Finger Domains Family (UHRF), which maintains DNA methylation by binding to DNMT1, ensuring the propagation of methylation patterns during DNA replication [15]; and the Zinc Finger (ZNF) proteins family, with members like Kaiso, ZBTB4, and ZBTB38 that repress transcription by binding to methylated CpG sites, similarly to MBD proteins [16]. These mechanisms and protein families collectively regulate gene expression through DNA methylation, underscoring its vital role in maintaining cellular functions and epigenetic inheritance.

### 2.2. Histone Modification Regulation

Chemical modifications of histones play a key role in altering the chromatin structure by changing the interaction affinity between histones and DNA double strands. Post-translational modifications of histones are recognized as powerful regulators of gene expression and are strongly implicated in the onset of several human diseases [4,5]. Convincing evidence has demonstrated, among other things, that epigenetic signals can promote the onset of phenotypic changes by modulating the expression of genes that control cardiovascular homeostasis. These changes can reverse upon the cessation of various pathological stimuli and, above all, may be amenable to pharmacological intervention. New strategies for the development of innovative therapies are aimed at targeting epigenetic factors by recognizing and introducing or removing modifications at the DNA or histone level. The possibility of pharmacologically intervening on epigenetic changes to prevent or revert diseases is very fascinating and is receiving more and more attention. The modifications that occur at the histone level include different processes such as methylation, acetylation, phosphorylation, adenylation, ubiquitination, and adenosine diphosphate (ADP) ribosylation, which are all catalyzed by specific and highly regulated enzymes. Among these modifications, histone methylation and acetylation are the most widely studied and best understood [17]; therefore, our attention will focus on them.

#### 2.2.1. Histone Methylation

The chemical modification of histones through methylation is one of the most significant and extensively studied post-translational modifications involved in the regulation of chromatin structure and gene expression. Histone methyltransferases (HMTs) regulate histone methylation by transferring methyl groups from the donor coenzyme S-adenosyl-L-methionine (SAM) to lysine or arginine residues on histones. The primary methylation site is the N atom (ε-amino group) of the lysine (K) side chain. Common methylation sites on histone H3 include H3K4, which is associated with gene activation, and H3K9 and H3K27, which are linked to gene silencing [18]. The regulation of gene expression by lysine methylation is relatively stable. Gene activation is often associated with arginine methylation on histones H3 and H4, while gene silencing is connected to arginine demethylation. H3K4 methylation is enriched in transcriptionally active regions such as transcription start sites, promoters, and enhancers. Specifically, H3K4me1 is concentrated in enhancers, correlating with either active (H3K27ac) or inhibitory (H3K27me3) enhancer regions. H3K9 methylation, particularly H3K9me2 and H3K9me3, modulates gene repression through the formation of heterochromatin [19]. Considering this, it is highly likely that lysine and arginine methyltransferases and demethylases serve as molecular transducers of metabolic signals to chromatin, thereby influencing gene expression [20].

#### 2.2.2. Histone Acetylation

Acetylation can regulate gene transcription by altering histone charges and interacting proteins. Histone acetylation, orchestrated by histone acetyltransferases (HATs) and HDACs, primarily occurs at conserved lysine residues in the N-terminus of histones H3 and H4 [21]. The HDAC family is divided into four classes based on structural similarities and substrate specificities. Class I HDACs, which include HDAC1, 2, 3, and 8, are RPD3-like proteins. Class II HDACs are further divided into Class IIa (HDAC4, 5, 7, and 9) and Class IIb (HDAC6 and 10). Class III HDACs, known as sirtuins (SIRT1-7), are nicotinamide adenine dinucleotide (NAD+)-dependent deacetylases. Class IV consists solely of HDAC11. HDACs are notably implicated in pathological processes such as inflammation, cardiac hypertrophy, and HF [22,23]. Numerous lysine residues in histones can be acetylated, such as H3K4, H3K9, and H3K27. Lysine acetylation regulates protein function by altering the protein structure or binding affinity with other partners. These modifications are involved in a variety of pathologies, ranging from cancer to CVD [24,25].

### 2.3. Non-Coding RNA Regulation

Non-coding RNAs (ncRNAs) are transcribed from the genome, but they are not translated into proteins. Unlike messenger RNA (mRNA), transfer RNA (tRNA), and ribosomal RNA (rRNA), ncRNAs function directly at the RNA level within the cell, utilizing the informational content of their nucleotide sequences. These ncRNAs include small nuclear RNA (snRNA), small nucleolar RNA (snoRNA), microRNAs (miRNA), and other RNAs with known or unknown functions [26]. ncRNAs can be categorized based on their length and mechanisms of action, exerting epigenetic modulation through various pathways: ncRNAs less than 50 nucleotides include miRNAs, which complement mRNA and promote mRNA degradation or silencing, and small interfering RNAs (siRNAs), which silence gene expression; ncRNAs of 50–500 nucleotides comprise snoRNAs, which modify rRNA, and snRNAs, which can combine with protein factors to form small nuclear ribonucleoprotein particles involved in mRNA splicing; ncRNAs greater than 500 nucleotides include long ncRNAs (lncRNAs), which act as endogenous sponges for mRNAs and miRNAs, regulating gene expression, and circular RNAs (circRNAs), rich in miRNA binding sites, that act as miRNA sponges, thus relieving the inhibitory action of miRNAs on target genes and increasing their expression levels; circRNAs can also serve as templates for protein synthesis [27]. miRNAs are key players in the regulation of gene expression with their transcription levels being controlled by tissue-specific epigenetic modifications [28]. Recent studies have shown that miRNAs are crucial in the progression of several CVD, such as cardiac hypertrophy and myocardial fibrosis, and they could be utilized as therapeutic targets for treating these conditions [29,30]. Similarly, lncRNAs have been found to play important roles in CVD and blood diseases by regulating gene expression with high functional specificity [31]. Moreover, the involvement of circRNAs in the progression of CVD and autoimmune diseases suggests their potential use as clinical strategies for diagnosing and treating these diseases [32,33,34,35].

## 3. Epigenetics in Cardiovascular Diseases (CVD)

**Figure 2:**
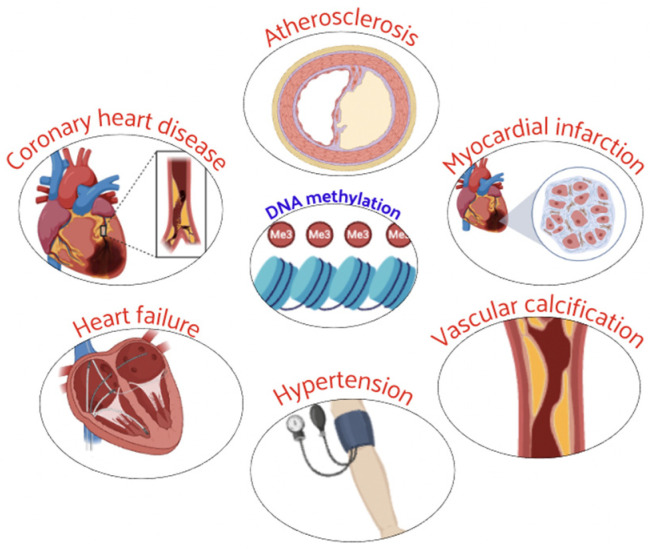
Abnormal DNA methylation plays a critical role in the development and progression of several CVD.

Epigenetics was identified to be correlated to CVD through the function and expression of epigenetic-related enzymes [36]. In particular, DNA methylation was shown to be associated with the expression of candidate genes related to CAD, HF, hypertension, and other CVDs. Their abnormal methylation status was involved in the CVD development (Figure 2) and can be used as a diagnostic marker of CVD progression [36,37].

DNA methylation is associated with CVD and incident CAD (iCAD) risk with implications for improving clinical risk prediction [38]. Recently, an association between differentially methylated regions and atherosclerosis was identified [39]. In acute myocardial infarction (AMI), the most critical stage was found to be at 6 h with a large number of methylation sites involved. Several genes (Ptpn6, Csf1r, Col6a1, Cyba, and Map3k14) were shown to be regulated in the AMI process by DNA methylation, and these genes are expected to be key markers for early diagnosis of AMI [40].

Crossing DNA methylation and gene expression data, recent approaches provided new insights into the CVD pathogenesis [41]. DNA methylation sites were identified to be associated with AMI in CAD [42], cardiovascular events, and high blood pressure [43,44]. These differentially methylated genes are present in pathways involved in lipid metabolism and inflammation related to the pathogenesis of CAD and AMI [45,46].

The role of DNA methylation in cardiac hypertrophy and HF is attracting much interest. Distinct regions of differential methylation, present in hypertrophic obstructive cardiomyopathy, dilated cardiomyopathy, ischemic cardiomyopathy, and chronic Chagas disease cardiomyopathy, reveal the role of DNA methylation in regulating HF-related genes in different clinical causes [47]. These differentially expressed and methylated genes in HF may serve as markers for detecting and diagnosing HF [48,49].

DNA methylation-related molecules are considered potential biomarkers for diagnosing vascular calcification, playing an important role in hypertension development [50,51,52]. The methylation level of several genes was significantly lower in hypertensive patients than in the control group, contributing to the development of hypertension [53,54,55]. These studies provide evidence that DNA methylation is closely related to CVD onset.

**Figure 3:**
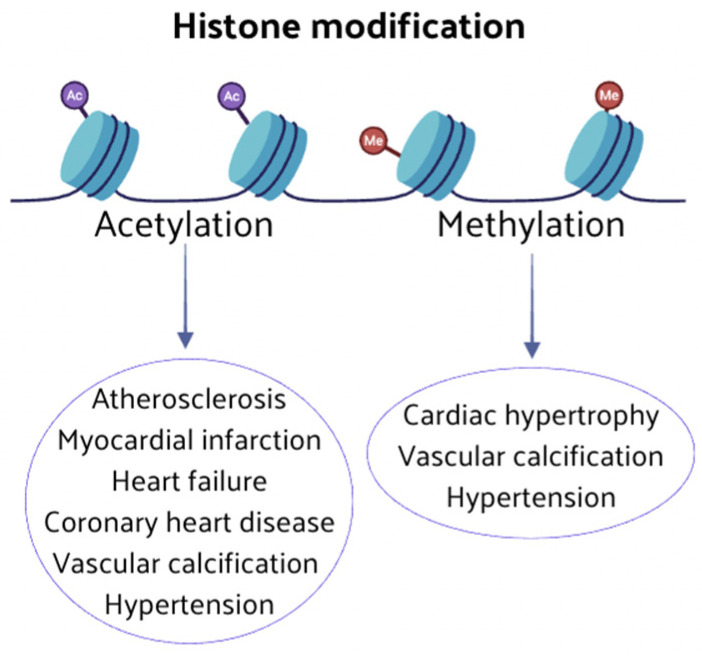
Abnormal histone modifications can induce changes in cellular phenotypes and cardiac function. Key enzymes involved in histone modification, such as methylation and acetylation, play pivotal roles in the onset and progression of CVD.

An imbalance in the expression of genes associated with CVD, resulting from an abnormal histone modification (methylation and acetylation), produces changes in cellular phenotypes and cardiac function. Key enzymes of histone modification may lead to the onset and progression of CVD by influencing cardiovascular pathophysiological pathways (Figure 3).

HMTs are essential for maintaining appropriate gene expression in normal cardiomyocytes and driving changes in the expression of genes associated with cardiac hypertrophy [56]. Histone methylation enhances osteoblast and vascular differentiation [57], and it is strongly associated with CVD physiopathologic mechanisms. HATs and HDACs are essential players in regulating histone acetylation and are involved in many epigenetic processes of vascular homeostasis and CVD. Research has shown that HDACs are involved in forming atherosclerotic plaques, inhibiting or promoting atherosclerosis [58,59,60,61]. Histone acetylation can attenuate myocardium ischemia–reperfusion injury and myocardial infarction [62,63].

HF is characterized by the apoptosis of myocardial cells, increased fibrotic scar tissue, and pathological myocardial hypertrophy, and many studies suggest that histone acetylation can regulate myocardial cell fibrosis in HF [64,65,66]. Pathological cardiac remodeling in HF is associated with myocardial apoptosis, and there is evidence that histone acetylation regulates the proliferation and apoptosis of cardiomyocytes [67,68,69,70].

**Figure 4:**
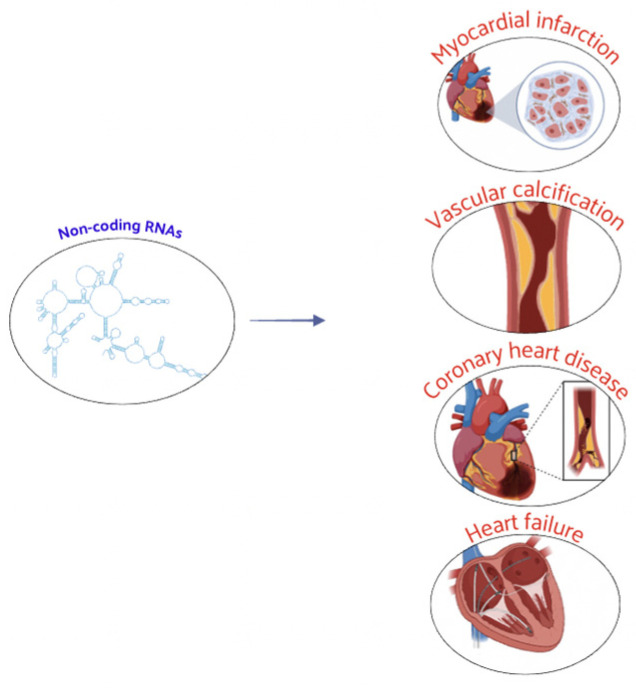
ncRNAs play a crucial regulatory role in CVD, influencing both physiological processes and pathophysiological mechanisms associated with CVD.

Further data have also accumulated on histone acetylation in the vascular calcification process. Studies have illustrated the potential of HDACs, such as SIRT6, HDAC9, and HDAC4, in treating vascular calcification, providing new targets and intervention strategies for clinical prevention and treatment [71]. A large number of studies has shown that ncRNAs play a key regulatory role in CVD. The identification of specific ncRNAs offers new directions for the early diagnosis and better prevention of these diseases. Nowadays, ncRNAs were associated with many physiological and pathophysiological processes of CVD, including CAD, myocardial infarction, and vascular calcification (Figure 4).

These ncRNAs, present in body fluids, are expected to become novel biomarkers in assessing the risk stratification, diagnosis, and prognosis of CVD. miRNAs can regulate the pathophysiological processes of CVD, being implicated in the development of CAD and acute coronary syndrome [72,73,74,75,76,77]. Recent studies have confirmed that exosome miRNAs are involved in myocardial remodeling and HF [78]. miRNAs are anticipated to be new tools for the diagnosis and treatment of HF. They are strongly associated with the onset and development of vascular calcification [62,79] and are important for regulating the pathogenesis of arterial hypertension [79,80]. lncRNAs act as both positive and negative regulators in CVD progression. They can reprogram cardiac fibroblasts into cardiomyocytes, activate cardiomyocyte differentiation, and participate in cardiac development [35,81,82,83]. lncRNAs can regulate atherosclerosis by interfering with the inflammatory responses, apoptosis, autophagy of vascular endothelial cells, foam cell formation, and lipid metabolism [44,84,85,86,87,88]. They are early indicators of myocardial infarction [5] and play crucial roles in pathogenesis by controlling autophagy, apoptosis, and other processes [89,90]. lncRNAs are involved in cardiac repair and functional development after myocardial infarction by regulating cell proliferation [89,91], suggesting new therapeutic targets. Further evidence shows lncRNAs involvement in HF progression, vascular calcification, and hypertension development [92,93,94]. circRNAs have gained attention for their role in CVD regulation. They are potential clinical markers for diagnosing CAD and myocardial infarction [51]. circRNAs contribute to atherosclerosis development by regulating vascular smooth muscle cell proliferation and migration, playing key roles in cardiac regeneration, repair, and apoptosis [90,95,96,97,98,99,100]. They are novel regulatory factors in cardiomyocyte hypertrophy, fibrosis, autophagy, and apoptosis, which are involved in HF development and may serve as diagnostic markers for vascular calcification and hypertension [101,102,103,104,105,106].

## 4. Epigenetic Modulation as Therapeutic Strategy for Cardiovascular Diseases (CVD)

### 4.1. DNA Methylation-Related Drugs

**Figure 5:**
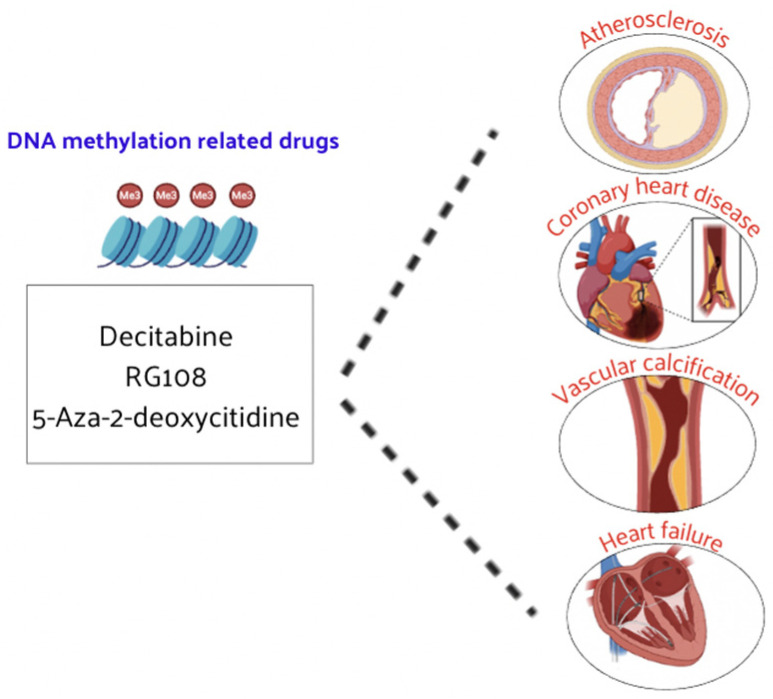
DNMT inhibitors have demonstrated therapeutic efficacy in CVD.

DNA methylation plays a crucial role in regulating gene expression and has significant implications in CVD such as CAD and HF. The reversible nature of DNA methylation makes it a promising target for therapeutic interventions. While research on DNA methylation-based therapies for CVD is still in its developmental stages, there is growing evidence supporting their potential efficacy. Decitabine has demonstrated therapeutic effects on atherosclerosis [107,108] and has also shown promise in reducing vascular calcification [109] (Figure 5).

Other DNMT inhibitors, such as RG108 and 5-aza-2′-deoxycytidine, have been found to slow the progression of atherosclerosis, CAD, and HF [110,111]. Large-scale clinical trials of these inhibitors are expected to be carried out in the future to prove their efficacy and safety in patients with CVD. These drugs could hold promise for the treatment of CVD, as DNA methylation inhibitors can alter the methylation status and expression levels of certain genes, leading to corresponding biological effects [111,112]. The influence on DNA methylation of epi-drug effects in pharmacology is being explored, providing a new perspective for understanding and treating CVD. Currently, DNA methylation-related drugs for various CVDs are still in the development phase and need to be further explored at a deeper level. For example, high methylation levels of the ABCA1 gene are associated with CAD and aging. Treatment with acetylsalicylic acid (ASA) can decrease the DNA methylation level of ABCA1, decreasing the occurrence of atherosclerosis and CAD [113].

### 4.2. Histone Modification-Related Drugs

**Figure 6:**
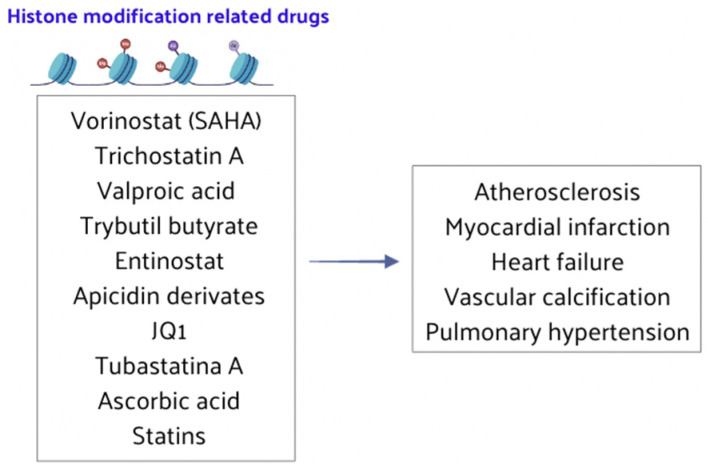
HMT and HAT/HDAC inhibitors play crucial roles in the treatment of CVD.

Recent research findings indicate that HMT and HAT/HDAC inhibitors are still scarcely used in the clinical treatment of CVD. Nonetheless, drugs targeting histone methylation and acetylation have shown promising effects in basic experimental research for treating CVD (Figure 6). In the next future, these drugs will be increasingly tested in clinical trials to address the symptoms and prognosis of CVD patients.

SMYD4, a member of the lysine methyltransferase family, also regulates histone acetylation by interacting with HDAC1. As a critical epigenetic regulator of heart development, SMYD4 has potential therapeutic applications in embryonic development and cardiogenesis [114]. The upregulation of SUV39H1, a histone methyltransferase that increases methylation of H3K9, significantly reduces infarct size and myocardial injury after ischemia–reperfusion injury. Thus, SUV39H1 represents a promising therapeutic target for managing ischemia–reperfusion injury, particularly in diabetes mellitus, and may intervene in endothelial dysfunction [115,116].

Histone acetylation can play an important role in treating atherosclerosis. Statins have been shown to partially restore overall HDAC activity, significantly impacting atherosclerosis pathogenesis [117]. Trichostatin A, a reversible and specific HDAC inhibitor, blocks the upregulation of markers associated with endothelial dysfunction and reactive oxygen species (ROS) in uremic conditions, making it a potential treatment for atherosclerosis and CAD [118]. HDAC inhibitors are also considered potential therapies for myocardial infarction and ischemia–reperfusion injury. For instance, Entinostat (MS-275), a Class I-specific HDAC inhibitor, significantly reduces myocardial infarction size and improves left ventricular function and tissue vitality, thereby protecting cardiac systolic function after ischemia–reperfusion [119,120]. Trichostatin A has also been found to increase myocardial cell formation and cardiac microvessels, reduce myocardial infarction size, and offer protective effects in myocardial infarction patients. This indicated that HDAC inhibition can preserve cardiac function and reduce cardiac remodeling by stimulating the endogenous cardiac capacity of regeneration [121,122,123,124]. Other HDAC inhibitors such as valproic acid, tributyl butyrate, and vorinostat (or suberoylanilide hydroxamic acid, SAHA) also showed efficacy in attenuating myocardial infarction size and ventricular remodeling by inhibition of HDAC along with inducing an increased angiogenic response [125,126,127]. Long-term, low-dose SAHA treatment has demonstrated lasting cardiac protection and no toxic effects, making it a potential candidate for clinical trials targeting myocardial infarction [128]. Vorinostat has shown efficacy in delaying ischemia–reperfusion injury in animal models, highlighting its potential for the future clinical treatment of such injuries [129].

Phosphodiesterase 5 inhibitors, such as sildenafil and adiponectin, protect the myocardium by increasing SIRT1 activity, improving myocardial function in diabetic mouse models [130,131,132]. Thus, targeting SIRT is a promising approach for developing CVD therapies.

HDAC inhibitors are vital for preventing and treating HF, offering protective effects on the heart [133,134]. Class I and II HDAC inhibitors, such as trichostatin A and apicidin derivatives, were reported to inhibit cardiomyocyte hypertrophy, improve cardiac function, and block cardiac remodeling [135,136,137,138]. These inhibitors also reversed myocardial fibrosis in HF by reducing fibroblast activation and inducing cell cycle arrest and apoptosis, showing clear clinical significance in treating myocardial diseases [139,140,141]. Furthermore, the histone acetylation reader BRD4 undergoes genome-wide stimulus-dependent redistribution in cardiac fibroblasts, suggesting that the BRD4 inhibitor JQ1 could be used for the treatment of HF and myocardial infarction in the future [142].

Epi-drugs and new strategies related to histone acetylation are crucial for advancing vascular calcification treatment. Inhibitors of p300 HAT reduce acetylated H3 and H4, alleviating aortic valve calcification, making p300 inhibition a potential therapeutic target [143,144]. HDAC6 expression is significantly decreased in aortic valve tissues of patients with aortic stenosis, and its downregulation may promote aortic valve calcification, positioning HDAC6 as a novel target for preventing and treating vascular calcification [145]. The HDAC inhibitor vorinostat is also a promising drug for treating vascular calcification [146].

HDAC inhibitors can serve as innovative therapy for pulmonary hypertension. The inhibition of HDAC6 reduces the proliferation and anti-apoptotic abilities of pulmonary smooth muscle cells, ameliorating pulmonary hypertension, representing a new therapeutic strategy [147]. Endothelial dysfunction is a critical determinant in hypertension and its complications, and histone acetylation plays an important role in controlling endothelial function. SIRT6, a member of the NAD+-dependent deacetylase (Class III HDAC), ultimately prevented hypertension and its complications, making SIRT6 a new therapeutic target for hypertension [148]. Tubastatin A (TubA), a selective HDAC6 inhibitor, could prevent hypertension progression [149], and ascorbic acid has shown efficacy in preventing hypertension occurrence and development [150]. These findings highlight new potential targets for antihypertensive therapies to serve as early prevention or treatment of CVD.

In summary, while statins are histone acetylation inhibitors with demonstrated efficacy in treating atherosclerosis and CAD, other HDAC and HAT inhibitors have shown improvement in the progression of atherosclerosis, CAD, and HF, but these have not yet been applied clinically for CVD treatment. Despite some HDAC inhibitors being used clinically for tumors, their application in CVD treatment remains limited due to the broad spectrum of HDAC substrates and potential side effects. Therefore, further exploration of the epigenetic regulation mechanisms in CVD development is necessary. Developing epi-drugs, related to the histone modifications, with high specificity and minimal side effects for CVD will be a key focus for future research efforts.

### 4.3. Non-Coding RNA-Related Drugs

**Figure 7:**
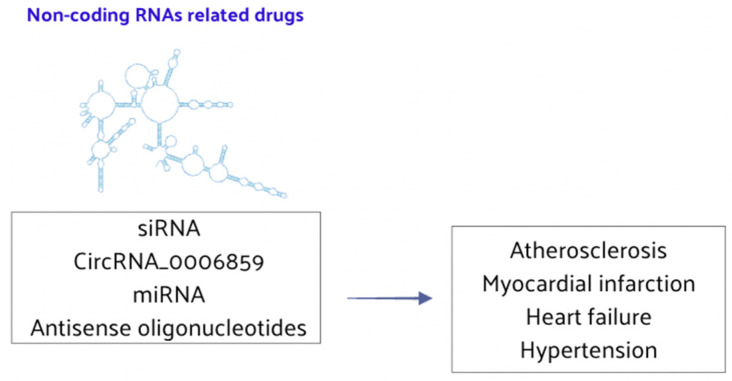
ncRNA-related drugs play a significant role in the regulation of CVD.

Recent evidence has highlighted the significant role of ncRNAs in CVD gene regulation and pathogenesis. Given their functional versatility, ncRNAs have emerged as potential targets for innovative clinical interventions. The rapidly advancing field of gene therapy, which includes antisense oligonucleotides (AOs) and small interfering RNA (siRNA), underscores this potential. These ncRNAs analogs or inhibitors exhibit low cytotoxicity when transfected in vivo, making them promising therapeutic agents for CVD. ncRNAs could serve as pivotal therapeutic targets in atherosclerosis. For instance, RNA interference (RNAi) therapeutic agents have been shown to reduce atherosclerotic lipoprotein levels and slow the progression of atherosclerosis [151]. Furthermore, lncRNAs of Small Nucleolar RNA Host Gene 1 (SNHG1) are highly expressed in vascular endothelium but decrease with disease progression. Its expression is negatively correlated with DNA damage and aging markers, suggesting that targeting miRNA-33 inhibition could play a significant role in treating hyperlipidemia [152,153]. The therapeutic potential of ncRNAs in myocardial infarction is also gaining attention. Additionally, certain drugs can regulate ncRNAs to treat HF, offering new strategies for delaying cardiac aging and providing myocardial protection [154,155]. ncRNAs are poised to become novel therapeutic genes for treating vascular calcification. lncRNAs have been identified as critical regulators of osteoblast differentiation, presenting a strategic opportunity for vascular calcification treatment [48,156]. Specifically, circRNA_0006859 may prevent vascular calcification, serving as an effective therapeutic gene [157]. These findings suggest that RNA therapy is a promising strategy for managing CVD (Figure 7).

ncRNAs play essential regulatory roles in complex biological processes and are potential therapeutic targets for various CVDs. Anti-miRNAs and AOs inhibit specific miRNA expressions to modulate the occurrence and progression of CVD, and such strategies are already being employed in clinical treatments. By targeting ncRNAs, it is possible to enhance the status and function of cardiomyocytes and vessels, representing a novel approach to CVD treatment. Future clinical work should focus on detecting cardiovascular-related ncRNA plasma levels to assess disease presence and severity. Moreover, gene-targeted therapies could potentially revert pathological changes in CVD, improving patient prognosis. Currently, epi-drug clinical trials and studies are focusing attention on atherosclerosis, CAD, HF, hypertension, myocardial infarction, and other CVDs. Nevertheless, most research involving epigenetic regulation in CVD remains in the preclinical or early clinical trial stages. Although epi-drugs are not yet widely used in clinical practice for CVD [158], ongoing investigation and large-scale clinical trials are expected to develop new applications. These advancements will aim to ameliorate symptoms and improve the prognosis for patients with CVD.

## 5. Epigenetic Dietary Components in Cardiovascular Diseases (CVD)

Identifying the direct epigenetic effects of individual natural compounds and food components, and ascertaining causal and consequential relationships in epigenetic biomarkers, remains a significant challenge of scientific research. Diet plays a crucial role in epigenetic intervention for the CVD prevention and treatment. The timing of exposure to ENCs significantly affects the extent of the epigenetic impact, particularly during critical periods such as early gestation [159]. Correlations between changes in maternal diet, epigenetic perturbations during uterine development, and the development of CVD have been widely documented [159,160,161]. Studies have shown that exposure to prenatal famine during early gestation is correlated with a higher risk of CAD and hypertension in adulthood as well as altered DNA methylation within the INSR and CPT1A loci, which encode proteins involved in prenatal development and fatty acid oxidation [159,160,161]. An excessive intake of certain dietary components has been linked to the pathogenesis of CVD, metabolic syndrome, and insulin resistance [8]. High-carbohydrate and lipid diets can increase acetyl-CoA levels, induce chromatin structure changes, and suppress autophagy, accelerating age-associated pathologies [162]. Changes in DNA methylation sites of genes such as APOE, IL6, and ABCA1 related to CVD traits are influenced by circulating fatty acids such as α-linolenic acid, EPA, and DHA. High-fat diets have been associated with increased DNA methylation in genes involved in adipocyte differentiation and lipid metabolism [163,164]. A high-fat diet induces differential methylation patterns in genes regulating lipogenesis and lipoprotein metabolism. Saturated fatty acids cause variations in the methylation levels of proinflammatory signals compared to polyunsaturated fatty acids [164]. Hyperglycemia in vascular and inflammatory cells triggers chromatin changes, influencing gene transcription [165,166].

Fasting and calorie restriction have opposite effects on overnutrition, resulting in increased DNA methylation and chromatin accessibility at SIRT gene transcription start sites [167]. In other studies, calorie restriction has been linked to a reduced risk of CVD, likely due to its effects on weight and adiposity, and transient beneficial effects on lipid profiles, although it does not affect the DNA methylation patterns of the *Fasn* gene [168,169].

The epigenetic effects of “methyl donor” nutrients on vascular aging and cardio-metabolic risk are well documented. Dietary intake of methyl group donors and cofactors during pregnancy influences fetal growth and development, linking early environmental exposure to chronic disease development in offspring [170,171]. Methyl groups from methionine, choline/betaine, and folate/vitamin B12 directly influence DNA and histone methylases, as they are precursors of SAM [172]. It is known that the maternal diet and supplementary intake of methyl group donors during the periconceptional period increase the DNA methylation of genes related to growth, metabolism, and appetite control, positively impacting cardiovascular health [173]. Dietary intake of carotenoids and vitamins B is associated with longer telomeres, which is a condition linked to a lower risk of developing CVD [174,175,176]. Dietary components have a profound impact on epigenetic mechanisms, as summarized in Table 1, and these effects significantly influence cardiovascular health.

Increased leukocyte telomere length (LTL) and reduced LINE-1 methylation were observed in elderly subjects after one year of vitamin B supplementation [177]. Shorter LTL is associated with lower folate levels, which is possibly due to the decreased DNA methylation of subtelomeres and telomere integrity loss [178,179].

Dietary components exert significant epigenetic effects that influence cardiovascular health. Understanding these mechanisms and their timing is crucial for developing effective dietary interventions to prevent and treat CVD. Further research is needed to elucidate the complex interactions between diet, epigenetics, and CVD.

### Epigenetic Natural Compounds (ENC) in Cardiovascular Diseases (CVD)

**Figure 8:**
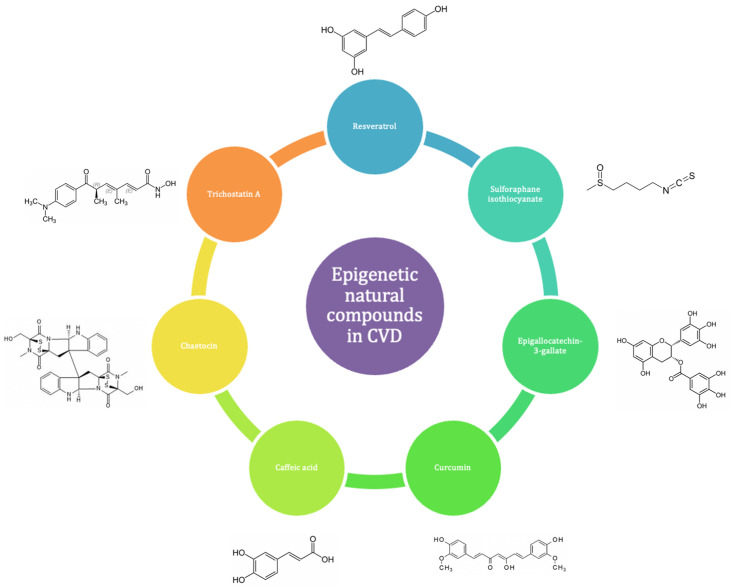
ENCs have demonstrated positive effects in both the prevention and progression of CVD.

Many bioactive natural compounds have been studied for their potential role in the prevention and treatment of CVD (Figure 8).

Polyphenols, including flavonoids, curcuminoids, and stilbenes found in fruits, vegetables, and other food derivatives such as green tea, red wine, and cocoa, form a group of bioactives with well-documented epigenetic and cardioprotective actions [8,180]. Several studies have confirmed their beneficial effects on vascular structure and function, inflammation, and multiple cardiovascular risk factors [181]. However, while their effects on the epigenome have been extensively studied in cancer, their role in the cardiovascular epigenome is still largely unexplored [181,182]. For example, resveratrol found in grapes, berries, peanuts, and red wine affects chromatin segregation [183] and activates SIRT deacetylases [184], impacting high glucose-induced cardiac oxidative stress, mitochondrial dysfunction, myocardial fibrosis, and vascular aging. A recent study on PBMCs from patients with type 2 diabetes showed that resveratrol supplements increased SIRT-1 expression, lowered H3K56ac levels, and alleviated oxidative stress [185]. Cruciferous vegetables are rich in the sulforaphane isothiocyanate, which is known to suppress NF-κB signaling and TNF-α-induced monocyte adhesion, circulating adhesion molecules, and chemokines in C57BL/6 mice [186]. Although cancer studies have shown that sulforaphane downregulates histone deacetylase activity and indirectly influences methylation [187], its role in the epigenetics of vascular diseases remains unexplored. Recent studies on other bioactive compounds have provided insights into their epigenetic effects. Dietary curcumin, the major curcuminoid in turmeric, suppressed extracellular matrix degradation following abnormal changes in the vasomotor tone of spontaneously hypertensive rats [188]. Curcumin-fed animals also showed decreased expression levels of HDAC1 and inflammatory markers matrix metalloproteinase-2 (MMP-2) and transforming growth factor β (TGFβ) in their coronary arteries as well as increased histone H3 acetylation at the TIMP1 promoter [188]. Similarly, epigallocatechin-3-gallate (EGCG), a catechin of green tea, induced H3 hypoacetylation and suppressed HDAC1 expression in endothelial cells, blocking inflammatory mediators [105,189]. Despite these findings, it remains unclear whether these epigenetic changes play a causal role in CVD or can serve as biomarkers for prevention and early intervention. Coffee extract and components such as caffeine, chlorogenic acid, and caffeic acid may impact gene expression by altering DNA methylation, histone modifications, and ncRNA expression. Coffee consumption during pregnancy has been linked to negative effects on offspring due to epigenetic changes, highlighting the need for further research to understand these mechanisms [190]. In a recent in vitro study, cocoa polyphenols downregulated key genes (DNMT, MTHFR, and MTRR) involved in the epigenetic process in PBMCs [191]. The dried root of danshen (*Salvia miltiorrhiza* Bunge) inhibits the JMJD2A methyltransferase and reduces H3K9 trimethylation, potentially exerting protective effects on HF [91,192].

The epigenetic actions of bioactive compounds or natural extracts and their potential applications in HF are summarized in Table 2.

A comprehensive understanding of these epigenetic effects is crucial for developing effective dietary interventions to prevent and treat CVD. However, further research efforts need to be undertaken to better understand the relationships between diet, epigenetics, and CVD.

## 6. Epigenetic Natural Compounds (ENCs) as Potential Therapeutic Interventions in Cardiovascular Diseases (CVD)

Epigenetic mechanisms such as DNA methylation, histone modification, and ncRNA regulation play pivotal roles in the CVD pathogenesis. Recent advancements highlight the therapeutic potential of epi-drugs in managing CVD [193]. This has stimulated research into natural compounds capable of targeting these mechanisms to mitigate CVD.

DNA methylation, governed by enzymes like DNMTs, regulates gene expression by modifying chromatin structure. Natural DNMT inhibitors, such as cocoa extract, have demonstrated efficacy in preclinical models by reducing DNMT levels and MTHFR expression, thus lowering atherosclerosis and CAD risk [191]. Clinical studies further support these findings, showing combined cocoa and statin therapy to decrease cholesterol levels, offering cardiovascular protection [191].

Histone modifications, including methylation and acetylation, profoundly influence gene expression and cardiac function. Inhibitors targeting HMTs and HDACs have shown promise in CVD treatment. For instance, chaetocin, a fungal metabolite, inhibits HMT SU(VAR)3-9, enhancing survival and mitochondrial function under cardiac stress conditions [194]. Danshen (*Salvia miltiorrhiza* dried roots) acts as an HMT inhibitor to mitigate cardiac hypertrophy and remodel left ventricular geometry through JMJD2A and H3K9 trimethylation pathways [91,192,195].

Natural compounds like resveratrol, found in grapes, and curcumin, from turmeric, modulate histone methylation and acetylation to confer cardiovascular benefits. Resveratrol reduces H3K27me3 levels, improving hypertension outcomes and promoting mitochondrial biogenesis to protect against ischemia–reperfusion injury [196,197,198]. Curcumin inhibits p300 HAT activity and histone acetylation, preventing cardiac remodeling and maintaining ventricular function in HF models [199].

EGCG from green tea acts as a DNMT and HAT inhibitor, modulating inflammatory gene expression, enhancing autophagy, and improving endothelial function through AMPK/mTOR pathways [200,201,202]. Sulforaphane, sourced from cruciferous vegetables, inhibits HDACs to prevent vascular remodeling and attenuate endothelial inflammation linked to conditions such as HF with preserved ejection fraction (HFpEF) [203,204,205,206]. Caffeic acid, a Class I and II HDAC inhibitor, modulates SIRT1 and SIRT3 to safeguard against cardiac dysfunction, oxidative stress, and adverse remodeling [207,208]. Epigenetic-related targets of principal natural compounds and their role in CVD are summarized in Table 3.

Despite promising results, challenges like lack of specificity and potential side effects remain in current epigenetic therapies for CVD. Future directions involve enhancing specificity through cell-targeted therapies and exploring RNA-targeted approaches for precise gene modulation [193]. Continued research into natural compounds with defined histone modification activities and minimal adverse effects holds promise for advancing personalized treatments and improving cardiovascular outcomes worldwide.

## 7. Conclusions

Understanding the intricate molecular mechanisms underlying CVD remains a frontier ripe for exploration in biomedical research. Recent years have seen an increasing recognition of the therapeutic potential of various epi-drugs in managing CVD. Notably, HDAC inhibitors have been extensively investigated for their ability to mitigate atherosclerosis, myocardial infarction, and HF. Similarly, inhibitors targeting histone methylation and acetylation pathways hold promise in treating conditions like CAD, myocardial infarction, and hypertension, despite ongoing challenges such as off-target effects that necessitate further refinement in their development.

ncRNA-based therapies, although promising, face obstacles including limited foundational evidence and the need for robust clinical validation. Epigenetics presents a promising avenue for both diagnosing and intervening in CVD. Recent studies have shed light on how natural compounds, nutrients, and dietary patterns can influence the epigenome, particularly through the modulation of histone acetylation and DNA methylation. Cardioprotective dietary regimens, such as the Mediterranean diet, can be related to epigenetic changes that regulate inflammatory and immune responses crucial in CVD pathophysiology. The flourishing field of epigenetics holds substantial promise for pioneering novel therapeutic strategies for CVD. Given the reversible nature of epigenetic modifications, genes and proteins governing these processes emerge as potential targets for innovative treatments across diverse cardiovascular disorders. The overarching goal is to develop epi-drugs with enhanced specificity, minimized side effects, and reduced risk of drug resistance. This evolving landscape underscores the transformative potential of epigenetics and genomics in reshaping treatment paradigms for CVD in the foreseeable future.

## Figures and Tables

**Table 1:** Epigenetic dietary components in CVD.

Dietary Factors	Epigenetic Changes	Biological Effects	Cardiovascular Risk	References
Excess in carbohydrates	Increase in the acetylation of DNA binding protein	Increase in acetyl-CoA, suppression of autophagy and acceleration of age-associated pathologies	↑	[159,161,162,165,166]
Excess in lipids	Increase/decrease in DNA methylation in some genes that regulate lipogenesis and lipidic metabolism i.e., APOA5, CPT1AVariation of methylation level of proinflammatory signals i.e., FTO, IL6	Altered inflammatory response	↑	[159,161,162,163,164]
Fasting conditions	Increase in DNA methylation and chromatin accessibility i.e., SIRT genes	Benefic effect on levels of total cholesterol, HDL and TG on weight and adiposity	↓	[167,168,169]
Methyl group donors (folic acid, B group vitamins)	Influence DNA and histone methylases, increase in DNA methylation of growth-related genes, LTL, LINE-1	Beneficial effect on metabolic function and appetite control with positive impact on cardiovascular health	↓	[170,171,172,173,174,175,176,177,178,179]

**Table 2:** Bioactive compounds: epigenetic actions and potential applications in CVD.

Natural Compounds and Total Extracts	Epigenetic Action	Potential Application for HF Prevention or Treatment	References
Resveratrol	Activates SIRT deacetylases, reduces H3K56ac levels	Reduces cardiac oxidative stress induced by high glucose, mitochondrial dysfunction, myocardial fibrosis, and vascular aging.	[183,184,185]
Sulforaphane	Suppresses NF-κB signaling, downregulates histone deacetylase activity, indirectly influences methylation	Reduces monocyte adhesion, circulating adhesion molecules, and chemokines.	[186,187]
Curcumin	Suppresses HDAC1 expression, decreases inflammatory markers (MMP-2, TGFβ), increases histone H3 acetylation	Reduces extracellular matrix degradation and inflammation in coronary arteries.	[188]
Epigallocatechin-3-gallate (EGCG)	Inhibits HAT, induces H3 hypoacetylation, suppresses HDAC1 expression	Blocks the response of inflammatory mediators in endothelial cells.	[189]
Coffee extract and components	Alter DNA methylation, histone modifications, ncRNA expression	Impact on gene expression and health outcomes.	[190]
Cocoa extract	Downregulates key genes involved in epigenetic processes (DNMT, MTHFR, MTRR)	Potential cardiovascular health benefits through regulation of epigenetic processes in peripheral blood mononuclear cells.	[191]
Danshen extract	Inhibits JMJD2A methyltransferase, reduces H3K9 trimethylation	Effects on heart failure.	[91,192]

**Table 3:** Epigenetic targets of principal natural compounds in CVD.

Compound	Epigenetic Action	Epigenetic Target	Disease	References
Resveratrol	Histone modification	H3K27me3, Class I, II and IV HDAC, SIRT1, IL6, FOXO 1	Hypertension, CAD, HF, atherosclerosis	[183,185,196,197,198]
Sulforaphane	Histone modification	Class IIa HDAC, HDAC2	Vascular remodeling, fibrosis, HF	[186,187,203,204]
Curcumin	Histone modification	HAT	Ventricular hypertrophy, CAD, HF	[188,199]
Epigallocatechin-3-gallate	DNA methylation, histone modification, non-coding RNA	DNMT, HAT, miRNA	HF	[105,189,202]
Chaetocin	Histone methylation	H3K9 methyltransferase	HF	[194]
Caffeic acid derivative	Sirtuins modulation	Class I and II HDAC	Cardiac dysfunction and hypertrophy	[207]
Trichostatin A	Histone modification	HDAC	Atherosclerosis, myocardial infarction, CAD, HF	[118,135,136,137,138]

## Data Availability

Data is contained within the article and related references.
